# Improvement of Working Memory and Processing Speed in Patients over 70 with Bilateral Hearing Impairment Following Unilateral Cochlear Implantation

**DOI:** 10.3390/jcm10153421

**Published:** 2021-07-31

**Authors:** Steffen Knopke, Arvid Schubert, Sophia Marie Häussler, Stefan Gräbel, Agnieszka J. Szczepek, Heidi Olze

**Affiliations:** 1 Department of Otorhinolaryngology, Head and Neck Surgery, Campus Virchow-Klinikum, Charité—Universitätsmedizin Berlin, 13353 Berlin, Germany; arvid.schubert@web.de (A.S.); sophia-marie.haeussler@charite.de (S.M.H.); stefan.graebel@charite.de (S.G.); 2 Department of Otorhinolaryngology, Head and Neck Surgery, Campus Charité Mitte, Charité—Universitätsmedizin Berlin, 10117 Berlin, Germany

**Keywords:** cochlear implantation, working memory, processing speed, cognition, WAIS-IV

## Abstract

Several studies demonstrated the association of hearing disorders with neurocognitive deficits and dementia disorders, but little is known about the effects of auditory rehabilitation on the cognitive performance of the elderly. Therefore, the research question of the present study was whether cochlear implantation, performed in 21 patients over 70 with bilateral severe hearing impairment, could influence their cognitive skills. The measuring points were before implantation and 12 months after the first cochlear implant (CI) fitting. Evaluation of the working memory (WMI) and processing speed (PSI) was performed using the Wechsler Adult Intelligence Scale 4th edition (WAIS-IV). The audiological assessment included speech perception (SP) in quiet (Freiburg monosyllabic test; FMT), noise (Oldenburg sentence test; OLSA), and self-assessment inventory (Oldenburg Inventory; OI). Twelve months after the first CI fitting, not only the auditory parameters (SP and OI), but also the WMI and PSI, improved significantly (*p* < 0.05) in the cohort. The presented results imply that cochlear implantation of bilaterally hearing-impaired patients over 70 positively influences their cognitive skills.

## 1. Introduction

According to the WHO, approximately 466 million people worldwide are affected by disabling hearing loss [1]. One-third of these people are over 65 years of age. In people over 70 years old, the number of hearing-impaired persons increases to about two-thirds of that population [2]. However, despite the increasing incidence of auditory disorders, only around 20% of those affected are adequately rehabilitated with hearing aids [3,4].

Hearing disorders contribute to functional and skill decline, and can lead to social isolation, depression, and reduced quality of life [5,6]. Furthermore, especially in the elderly, auditory disorders contribute to cognitive decline [7,8,9]. Thus, the importance of hearing impairment or hearing rehabilitation in the interaction between structural brain damage and neurocognitive deficits emerges as an essential field of study [10]. Furthermore, elucidating this interaction could influence the prevention of cognitive functional impairments, resulting in improved health economics [11].

Extensive epidemiological studies have already demonstrated that hearing disorders are associated with neurocognitive deficits and dementia [12]. Mild hearing impairment doubles the risk of developing dementia compared to the well-hearing population, whereas severe hearing loss increases the risk of dementia fivefold [2]. Furthermore, auditory disorders have been identified as the most critical, preventable factor contributing to the development of dementia [13,14].

Dementia affects predominantly, but not exclusively, older people. Dementia is a syndrome manifested by various organic brain disorders [14,15], disturbing memory, thinking, behavior, and the ability to carry out everyday activities [16]. Neurodegenerative processes resulting from the hearing impairment increase the sensory and perception-related limitations [11,17]. In addition, the processing of incomplete phonological information ties up a large part of cognitive attention [18]. The ability to compensate for these limitations is determined by working memory capacity and implementing processes in a time-dependent manner. In addition, it is variable and individually distinct [17,18,19].

Working memory and processing speed strongly correlate with fluid intelligence, which is responsible for abstract reasoning and problem-solving [20]. The fluid intelligence starts to decline from approximately the age of 50. Longitudinal epidemiological studies have shown that the most significant factor affecting processing speed and working memory is aging [21]. In contrast to research on the executive function of working memory, only a few studies dealt with the importance of processing speed in connection with hearing impairment. In particular, speech processing in background noise seems to be directly dependent on the processing speed, and could therefore be influenced by hearing rehabilitation [22,23,24].

Hearing disorders have only been given a subordinate role in the recent past. Today, almost all types of hearing impairment can be treated. Depending on the severity and localization of hearing impairment, rehabilitation of sensorineural hearing loss ranges from using a digital conventional hearing aid to cochlear implants (CIs). For the auditory rehabilitation of hard-of-hearing people, cochlear implants have been used successfully in various age groups, including the elderly, suggesting that advanced age is not a limitation for CI [25]. 

The scientific examination of hearing loss and its effects on the elderly is critical for healthy aging. Recent studies demonstrated that hearing impairment has consequences that are more far-reaching than the loss of communication skills. For example, rehabilitation with CIs reduced depression, anxiety, stress, and tinnitus [26,27,28,29,30,31,32]. Moreover, published reports have demonstrated changes in cognitive abilities after cochlear implantation [33,34,35,36,37,38]. In addition, numerous studies determined the link between hearing disorders and cognitive function [11,39,40,41,42,43,44,45,46,47,48,49,50,51,52,53].

Based on the link between hearing impairment and a significant increase in cognitive deficits, recent research in the field has focused on studying the association between auditory rehabilitation with CIs and dementia diseases [19]. In the literature, various screening methods for dementia and cognitive decline are used. However, there is a lack of data on older patients rehabilitated with CIs, which would determine the cognitive status using an internationally comparable and age-independent scaled instrument.

The present work aimed to describe changes in the cognitive performance of bilaterally hearing-impaired patients over 70 who underwent auditory rehabilitation with unilateral CIs. A standardized intelligence test, WAIS-IV, was used to determine cognitive performance [54] before and after implantation. The necessary age-related grading of the cognitive function allowed subsequent comparison with different age groups. 

## 2. Materials and Methods

The local ethics committee (permit number EA2/030/13) approved this prospective, non-interventional, and longitudinal study. All investigations were conducted according to the principles expressed in the Declaration of Helsinki. All patients have given their informed written consent. 

### 2.1. Inclusion Criteria

Patients of both genders were consecutively included in this study. The following inclusion criteria were used:

- Diagnosis of post-lingual, bilateral, and severe hearing loss with speech perception ≤ 60% in the Freiburg Monosyllabic Test (speech perception in quiet) and with best-fitted hearing aids with sound pressure level (SPL) 65 dB, as previously described [55]
- German mother tongue
- Age 70 and above
- Unilateral cochlear implant
- Meeting of the clinical criteria for cochlear implantation:
  ○ Possibility of using general anesthesia
  ○ Exclusion of retrocochlear disorder (e.g., vestibular schwannoma)
  ○ Unremarkable cochlear anatomy
  ○ Motivation for postoperative audiological rehabilitation

### 2.2. Exclusion Criteria

- Diagnosed dementia syndrome or mild cognitive impairment in the medical history
- Severe visual impairment in the medical history
- Lost to follow-up (e.g., severe general illness)

### 2.3. Explanation of the Number of Patients Included in the Study

On average, our center performs about 135 cochlear implantations annually; about 25% of the patients (33–34 subjects) are over 70 years old. Of them, 65% (21–22 subjects) are severely hearing impaired on both sides. Of these, about 20% (4–5 subjects) will have the second side implanted within a year after the first surgery, and cannot be evaluated further (for the purpose of this particular study). That means that every year, around 17 to 18 patients could be included. However, some do not want to participate in clinical studies; some do not meet the inclusion criteria; and some pass away from reasons unrelated to implantation or are lost to follow-up. In the end, we have about 5–10 patients a year who meet the inclusion criteria and are willing to participate.

### 2.4. Description of Study

**Figure 1:**
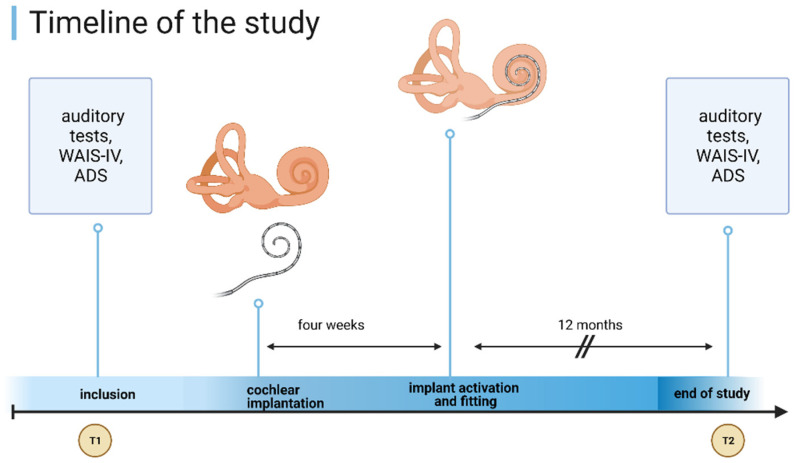
Study design and timeline. Examination point: T1 = inclusion into the study before CI and first evaluation; T2 = 12 months after first CI fitting and second evaluation. Created with BioRender.com (accessed on 9 June 2021).

Twenty-one patients met the inclusion criteria and were followed up with at least twelve months after unilateral cochlear implantation with a multi-channel cochlear implant produced either by MED-El^®^ (Synchrony, MED-El, Innsbruck, Austria) or by Cochlear^®^ (Nucleus, Cochlear, Sydney, Australia). The appointments were scheduled before surgery and twelve months after the first CI fitting (see Figure 1). The surgery took place in a university cochlear implant center between 2015 and 2018.

### 2.5. Audiological Assessment: Speech Perception (SP) in Silence and in Noise

The Freiburg Monosyllabic Test (FMT) [56] was used to determine the preoperative speech perception in silence at 65 dB SPL (sound pressure level) with best-fitted hearing aids. After implantation, the FMT was performed with the CI speech processor turned on. The FMT contains 20 groups of 20 phonetically similar monosyllabic words offered to the test subject in standardized conditions. Thus, a healthy person achieves 100% of speech understanding at 65 dB SPL. 

The Oldenburg Sentence Test (OLSA) was employed to determine the speech perception in noise [57]. The OLSA measures the speech intelligibility threshold in background noise at 65 dB SPL. The test was performed after surgery with a CI speech processor and masked contralateral ear. In detail, 20 test sentences per list were presented in a random combination with a fixed scheme (name, verb, number, adjective, and object). The signal-to-noise ratio (S/N), at which the correct word score is 50% (critical S/N), was achieved by adjusting the sound pressure level for each sentence depending on the response to each test item. In the present study, we applied the sound presentation configuration S0N0 (speech and noise from the front). The speech intelligibility threshold for a person with healthy hearing is −7.1 dB S/N (65 dB SPL) [57]. Lower values correspond to better speech perception. 

The audiological assessment was performed one, three, six, and twelve months after the first fitting of the CI. 

### 2.6. Audiological Rehabilitation

The speech processor was first fitted four weeks after the implantation. Then, as part of the follow-up rehabilitation, the setting was improved weekly by an experienced audiologist and a specialized speech therapist in a face-to-face situation. Thus, with increasing time intervals, twenty therapeutic sessions were completed over 12 months. Each therapy unit consisted of a speech therapy session and an audiological speech processor adaptation. Approximately 90 to a maximum of 120 min were spent per appointment. In addition, no cognitive training and no intermediate intelligence tests were performed during the auditory rehabilitation process.

### 2.7. Audiological Assessment: Oldenburg Self-Assessment Inventory (OI)

The OI comprises three subcategories: hearing in quiet, hearing with background noise, and sound source localization. The 12 closed questions about everyday situations are rated with points from 1 to 5. The higher the score, the better the subjective hearing [58].

### 2.8. Screening for Depressiveness: General Depression Scale–Long (ADS-L)

The ADS-L includes 20 items and allows the self-assessment of impairment by depressive symptoms in the last few weeks. The internal consistency (Cronbach’s α) ranges from 0.89 to 0.92; the cutoff value is 23 points [56]. The ADS-L was used to find out relevant depressive symptoms before and after surgery. The instrument has been validated for use in clinical settings and for evaluating treatment effects.

### 2.9. Cognitive Performance Test (Wechsler Adult Intelligence Scale 4th Edition: WAIS-IV)

A validated German version of the WAIS-IV test (Hogrefe Verlag GmbH & Co. KG, Göttingen, Germany) was used to assess cognitive abilities. The WAIS-IV is an internationally established, standardized, and validated instrument for measuring age-matched cognitive performance. All subjects were tested before implantation and 12 months after the first CI fitting. In order to avoid learning effects, no cognitive tests were performed in between. The test was carried out in the same order for each subject.

The WAIS-IV enables the assessment of the cognitive development status of a person by determining an overall IQ. The scoring allows the calculation of four IQ indicators: speech understanding and perceptual logical thinking for crystalline intelligence, and working memory (WMI) and processing speed (PSI) for fluid intelligence. The individual index values were calculated based on the mandatory subtests connected with the associated tasks. 

The present study focused on the index processing speed (PSI) and the index working memory (WMI) because of their association with hearing impairments and fluid intelligence described above. The subtests “Coding” and “Symbol Search” were used to determine the PSI, whereas “Digit Span” and “Arithmetic” were used for the WMI [59,60]. The WMI index measures attention, concentration, and working memory, whereas the PSI index determines the speed of mental and graphomotor processing [61].

The raw values recorded were scaled and then converted into age-correlated total values, with normalization to 100 points and a standard deviation of 15 points. This normalization followed a Gaussian distribution; 100 points indicated average intelligence. Deviations up or down indicated above- or below-average intelligence.

The test was conducted strictly according to the test procedure manual in a highly standardized manner. The examiner read the instructions out loud. Before commencing each test, the subjects were asked whether they correctly understood the test procedure. Next, the test provided exercises that allowed the determination of whether the test person understood the task. The manual also provided instructions if the exercise was conducted incorrectly or a certain number of tasks would not be completed. Lip-reading was allowed. In addition, the repetition of the task was allowed upon request.

Hearing-impaired persons can also use the WAIS-IV. The prerequisite is the use of personal, best-fitted hearing aids on both ears. The test was carried out in a bright, soundproof room by the same investigator under standardized conditions following the WAIS-IV implementation protocol. The internal consistency with the reliability coefficient alpha was α = 0.94 for WMI and alpha α = 0.90 for PSI [59,61]. Both the audiological and cognitive tests were performed in the same order for each subject.

### 2.10. Statistical Evaluation

For the statistical analyses, SPSS version 25.0 (IBM, Ehningen, Germany) was used. The WAIS-IV was evaluated using the original software version 2.1.0 to calculate the scaled scores and the age-related IQ index values. According to a Gaussian distribution, the age-related norm group of the WAIS-IV is defined with an average of 100 IQ points. The results are presented as the mean (MV) ± standard deviation (SD). The level of significance was set at 5%. A two-tailed Wilcoxon signed-rank test was used to compare the scores before and after implantation, as most of the dataset lacked a normal distribution. The effect size was calculated following the recommendations of Cohen for non-parametric tests [62]. Correlations were performed by computing Spearman’s rank correlation coefficient (r_sp_). Multiple linear regression could not be performed because the test requirements were not met.

The a priori power analysis for calculating the directed difference in WAIS-IV before and after surgery resulted in a sample size of 20 subjects, assuming a normal distribution (effect size d = 0.8; alpha error = 0.05; power = 0.95).

## 3. Results

### 3.1. Patient Data and Age Distribution

Ten female (47.6%) and 11 male (52.4%) subjects were evaluated. The mean age in the study sample was 77.1 ± 5.5 years. There was an average of 39.6 days between the inclusion in the study (and the WAIS-IV test) and implantation. The average duration of hearing impairment was 32.0 ± 22.3 years.

### 3.2. Audiological Assessment 

#### 3.2.1. Speech Perception (SP) in Silence (FMT)

Preoperatively, when measured in silence with a masked contralateral ear, SP on the operated ear was 6.0 ± 10.2% (range 0–40%), and that of the contralateral, better-hearing ear was 29.5 ± 20.8% (range 0–60%). Twelve months after the initial CI fitting, the FMT score with CIs and a speech processor was 57.4 ± 22.0%, indicating significant improvement compared to the preoperative baseline value (*p* < 0.01). Each ear was tested separately; the opposite ear was masked.

#### 3.2.2. Speech Perception (SP) in Noise (OLSA)

The OLSA was tested only postoperatively with the CI processor switched on. Twelve months after the first CI fitting, the MV was 2.1 ± 2.4 dB S/N (range −1.5 to 6.0 dB S/N).

#### 3.2.3. Audiological Self-Assessment: Oldenburg Inventory (OI)

There was a significant improvement in all domains tested 12 months after the first CI fitting compared to the preoperative baseline value (two-tailed Wilcoxon signed-rank test, Table 1).

### 3.3. Screening for Depressiveness: General Depression Scale–Long (ADS-L)

The mean value of the ADS-L before implantation was 11.3 ± 8.8 points. Twelve months later, the mean ADS-L score was 12.5 ± 10.2. There were no significant differences between the before and after values (*p* = 0.59). In addition, none of the patients scored above the critical value of 23 points, which indicated major depression.

### 3.4. Cognitive Performance Test: Wechsler Adult Intelligence Scale 4th Edition (WAIS-IV)

The IQ index PSI and the IQ index WMI improved significantly during the 12 months after the CI fitting (Table 2). The four subscales of both IQ questionnaires had an increasing trend (Table 2).

The cognitive analysis of PSI and WMI resulted in a medium effect size according to Cohen [62], and a relatively large effect size according to Gignac [63]. Therefore, based on the study design, assessing the effect size was done using the Gignac criteria.

Spearman rank correlation was used to test the relationships between variables. No significant correlations were found between the cognitive parameters and speech perception, the OI, or the ADS-L.

## 4. Discussion

The present study analyzed the cognitive function of patients over 70 with severe bilateral hearing impairment using a standardized intelligence test. The main aim was to determine the cognitive status of the patients on an age-adjusted and comparable basis. The second aim was to analyze the impact of unilateral rehabilitation with CIs on patients’ working memory and processing speed. The WAIS-IV was chosen to ensure comparability of the results regardless of the disease and age, and to create a basis for the subsequent evaluation of the study results via an age-adjusted calculation of cognitive parameters.

Here, we report for the first time the WAIS-IV IQ index values reflecting the working memory and processing speed of elderly patients with bilateral severe hearing loss before and after unilateral cochlear implantation and auditory rehabilitation. One year after the first CI fitting, we found a significant improvement in the working memory and processing speed (*p* < 0.05). In addition, in agreement with earlier studies [26,30,31,32,64,65], we observed significant benefits in terms of speech perception in silence and in noise and subjective hearing. However, speech perception and the depression index were not associated with the WAIS-IV scores (*p* > 0.05).

In the present study, the test subjects were interviewed before and after surgery. Examiner-dependent procedural deviations were minimized. The ratio (1.0:1.1) of female to male subjects was balanced. The depression index was low before and remained low after implantation; thus, the possible influence of depressive symptoms on cognitive performance was excluded [36]. According to the applicable criteria [55], all patients qualified for bilateral cochlear implantation. Consequently, unilateral implantation was performed on the more inferior hearing side.

The measurement of speech perception with the FMT goes beyond pure tone audiometry (PTA). Understanding monosyllabic words in silence (or even sentences in background noise) is a complex hearing situation, and requires cognitive performance [66]. Numerous attempts were made to find a mathematical relationship between PTA and speech perception [67,68]. Recent research has identified a significant correlation between the audiometric thresholds and speech in noise [69]. Older people in particular experience more problems with speech perception while having relatively good PTA, which is a result of declining sensory abilities [70].

Moberly et al. [71] suggested age as a predictor of unsatisfactory performance concerning speech perception among older people who underwent cochlear implantation. On the other hand, some other studies found no significant differences in speech perception after cochlear implantation in the elderly compared to younger people [34]. Heinrich et al. [72] reported that the selection of speech tests could determine the link between speech perception, cognition, and subjectively perceived hearing impairment. In the present study, no association was found between the WMI and PSI, measured with the WAIS-IV, the speech perception in silence or noise, and self-assessment of hearing abilities.

The measurement of intelligence is based on the findings of Caroll, Horn, and Cattell, and is known as the CHC model [72]. It paved the way for many current intelligence tests, such as the Wechsler Intelligence Test used in the present study. The latest fourth edition of the WAIS represents a further development of David Wechsler’s idea [54], and studies different subdomains of cognitive performance. Because of scaling and normalizing, the WAIS-IV enables the qualitative and quantitative assessment of cognition. However, the results obtained with the WAIS-IV cannot be compared to those acquired using simple dementia screening instruments, such as the Mini-Mental State Examination (MMSE), which are commonly used in studies of cognitive deficits connected with hearing impairment. In one such study, Lin et al. [43] reported significantly better MMSE scores in the control subjects than the hearing-impaired people over a six-year period. A different study using a 25-year follow-up period determined that the MMSE score for people with hearing loss who used hearing aids differed significantly from those who have not used hearing aids [35]. However, unlike our study, patients with severe bilateral hearing loss were not included in these analyses.

The link between hearing rehabilitation using CIs and cognitive performance was already entertained at the beginning of the CI era [73,74,75,76]. Vega [73] and Crary et al. [74] reported the first findings on the relationship between CIs and cognitive function in the late seventies and early eighties of the last century, respectively. The cognitive abilities of implanted patients measured with previous versions of the WAIS have changed in an age-dependent manner. Presently, the technical advancement of the hardware and software of multi-channel electrode array implants with situation-based coding strategies has introduced a significant improvement, especially in the outcome of auditory comprehension rehabilitation [73,74]. In addition, the expansion of user groups and the demographic change ocurring in the industrialized countries brought new challenges and, at the same time, the issue of cognitive limitations in old age [2]. However, the necessity to examine age clusters separately concerning hearing impairment is a recent finding [77]. The collection of data in age-spanning groups from adolescents to retirees makes data comparison more complex. A structured review of publications with narrow inclusion criteria is necessary to expand the knowledge in intelligence research [75].

More recently, Mosnier et al. [36] contributed significantly to understanding CI effects in the elderly. These authors used a sizeable psychometric battery (MMSE, five-word test, clock drawing test, verbal fluency test, d2 test of attention, and Trail Making Test parts A and B) in a multicenter study, and found an improvement of cognitive parameters six and 12 months after implantation. Furthermore, Mosnier et al. [78] reported cognitive recovery in a group of patients aged 65+ one and five years after implantation. The improvement was noted in patients with abnormal initial values or mild cognitive impairment before implantation, but not in patients with average cognitive baseline values, which contrasts with our study. Thus, the discrepancies in the study outcomes might be due to the different age groups and different tests used. Another essential difference is implementing a cognitive training program as part of audiological rehabilitation for at least six months in the cohort of Mosnier et al. Cognitive training was not carried out in the present work.

On the other hand, the WAIS-IV has sufficient sensitivity to determine changes even with an average starting value. We therefore consider that the WAIS-IV offers adequate statistical power to analyze small study cohorts that are common in clinical CI research. In addition, the IQ values in our study had a typical spread and average mean values for both working memory and processing speed, enabling subsequent comparisons with future studies. Nevertheless, impaired hearing function could potentially produce biased results. However, under identical testing conditions, obtaining comparable values would be expected.

The cognitive assessment performed during our study was deliberately repeated only one year after the first CI fitting to avoid possible learning effects, which could have occurred if the test was repeated only six months after the first testing session [54]. Nevertheless, a cognitive training effect cannot be ruled out solely through the audiological training program, and a potential distortion is conceivable. Since the hearing rehabilitation program is essential for improving speech perception, this potential confounder cannot be ruled out.

Völter et al. [79] studied a cohort of 50–84-year-old patients that included 20 patients over 65 for six months. The comprehensive assessment instrument [80] indicated a significantly favorable CI influence on cognitive abilities in 33 test subjects. In addition, there was a significant improvement in working memory (n-back test) in patients over 65, but the processing speed remained unchanged. In a follow-up study, Völter et al. [34] reported significant changes in the working memory and processing speed (n-back test; OSPAN; TMT A + B) twelve months after implantation. During that time, preoperative differences between mid-age and older groups decreased. Völter et al. also reported the influence of age on working memory, being more pronounced in older patients. The authors compared the mean age values and observed a difference in performance between older and younger patients.

Sarant et al. [81] selected the first 20 participants from a cohort of 59 subjects to evaluate their cognitive functions with the MMSE and the Cogstate Brief Battery before and after cochlear implantation. An improvement in the executive function of the male participants without tertiary education was noticed 18 months after CIs; the results of other participants remained unchanged.

The influence of age on cognitive abilities, especially fluid intelligence, has been described many times, and is regarded as a part of the physiological aging process [20]. The present study aimed to determine if improving speech perception with CIs increases age-adjusted intelligence indicators of working memory and processing speed in a cohort of elderly. In our study, the age-adjusted determination of indicators accounted for the influence of age. Thus, our results can be compared to the results of a healthy population.

Mertens et al. [38] reported a significant increase in the domain of attention in the RBANS-H tool one year after CI fitting. The authors performed a comparison and model calculation with a control group to exclude the practice effect. At the same time, they admitted that an ideal matching would be challenging because of varying listening situations and demographics, as well as ethical reasons. The RBANS tool was developed and validated as a screening tool for dementia [82]. Therefore, the fundamental consideration of a control group offering statistical value is irrefutable, but the assessment tool used should be validated for the expected effect. Although the WAIS-IV has been viewed as complex and too strenuous for the test subjects, WMI and PSI can be performed separately, as was done in the present study. Furthermore, we made no fundamental assumption about the poor cognitive performance of the hearing impaired compared to normal-hearing individuals. However, we assumed that reducing the burden of hearing impairment on cognitive processes after CIs could improve the cognitive abilities of hearing-impaired individuals [52].

Although Zhan et al. [37] reported improved cognitive performance after CIs using individual subtests of the Wechsler intelligence score, the authors performed the test six months after CIs (and not twelve, as in our study), which means that the learning effect could have possibly occurred. In addition, the age of the patients ranged from 42 to 89 years, which does not exclude age-specific changes, and makes a direct comparison with our study possible only to a small extent. Therefore, to our best knowledge, this is the first report on age-correlated cognitive WAIS-IV data from bilaterally hearing-impaired patients over 70 who underwent unilateral cochlear implantation and were tested one year later. The cohort for this study is relatively small; therefore, further work is recommended with larger groups to gain additional statistical power and better understand the possible predictors of cognitive abilities and their change after hearing rehabilitation.

## 5. Conclusions

The present study determined the effects of a single-sided CI on hearing rehabilitation outcome and cognitive skills in bilaterally hearing-impaired individuals over 70. In addition to improved auditory performance, a significant enhancement of cognitive performance regarding working memory and processing speed was noted. Both working memory and processing speed are essential in speech perception, both in silence and in background noise.

## Figures and Tables

**Table 1:** Mean values (MVs) of the Oldenburg Inventory (OI) subscales before and 12 months after CIs. The level of significance indicates the difference between the examination points. The results are shown as MV ± SD.

	Before Implantation	12 Months after Implantation	Level of Sig. Two-Tailed
OI-total	2.29 ± 0.60	3.02 ± 0.63	*p* < 0.01
OI-quiet	2.62 ± 0.75	3.37 ± 0.85	*p* < 0.01
OI-noise	1.81 ± 0.57	2.66 ± 0.65	*p* < 0.01
OI-localization	2.63 ± 0.99	3.03 ± 0.94	*p* < 0.05

**Table 2:** Scores of the WAIS-IV subscales and composite IQ scales before implantation and 12 months after the CI fitting. The level of significance indicates the difference between the examination points. The results are shown as MV ± SD. The effect size is defined as a quotient of the Z-score of the Wilcoxon test and the square root of the number of total subjects [62].

	Before Implantation		12 Months after First CI Fitting		Level of Significance	Effect Size
	MV ± SD	Range	MV ± SD	Range	Two-tailed	
**Subtest Scaled Score**						
Digit Span	22.9 ± 5.9	14–34	24.1 ± 5.5	15–38	*p* = 0.51	
Arithmetic	12.5 ± 3.4	5–18	13.3 ± 3.6	8–22	*p* = 0.12	
Symbol Search	19.3 ± 7.0	6–33	21.0 ± 7.1	7–38	*p* = 0.14	
Coding	46.8 ± 14.7	23–74	49.6 ± 16.9	20–82	*p* = 0.09	
**Composite Score**						
Working Memory	92.7 ± 15.2	63–120	98.1 ± 15.3	74–139	*p* < 0.05	r = 0.33
Processing Speed	97.2 ± 16.2	70–133	101.9 ± 15.8	70–126	*p* < 0.05	r = 0.35

## Data Availability

Data available on request.
